# [WITHDRAWN] Novel FOXM1 inhibitor STL001 sensitizes human cancers to a broad-spectrum of cancer therapies

**DOI:** 10.21203/rs.3.rs-3711759/v2

**Published:** 2024-01-11

## Abstract

The full text of this preprint has been withdrawn by the authors while they make corrections to the work. Therefore, the authors do not wish this work to be cited as a reference. Questions should be directed to the corresponding author.

